# Welcoming All Unicorns

**DOI:** 10.19102/icrm.2017.080401

**Published:** 2017-04-15

**Authors:** Brian Olshansky


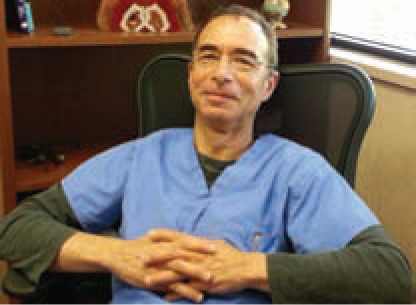


A unicorn is a mythical, legendary creature—a beast, with a single, pointed spiral horn thought to live on the edge of the earth in ancient times and to have the power to purify water and heal sickness. Here, we introduce this new section, *Case Unicorns* as an educational tool for electrophysiologists to help them improve their practice and assist them in treating their patients with greater insight. Our goal for this section is to present unique and challenging cases that provide mechanistic understanding of the electrophysiological properties and the nature of the condition, in order to help others deal with specific issues in their general practices. Our first unicorn involves a supraventricular tachycardia with two-to-one going to one-to-one atrioventricular conduction.

We hereby invite our readers to submit some of their most challenging and difficult cases, or cases that provide novel understanding and insight to our colleagues. Our hope is that these cases will be reviewed by some of the most prominent experts in the field to clarify the nature of the issue being addressed, and help them and others navigate some of the most challenging aspects of our profession. We hope you enjoy *Case Unicorns*.

Sincerely,

Brian Olshansky, MD, FHRS, FACC, FAHA

Professor Emeritus

University of Iowa

Cardiac Electrophysiologist

Mercy Hospital-North Iowa

Mason City, Iowa 50401

